# Identification of Thioredoxin Glutathione Reductase Inhibitors That Kill Cestode and Trematode Parasites

**DOI:** 10.1371/journal.pone.0035033

**Published:** 2012-04-20

**Authors:** Fabiana Ross, Paola Hernández, Williams Porcal, Gloria V. López, Hugo Cerecetto, Mercedes González, Tatiana Basika, Carlos Carmona, Martín Fló, Gabriela Maggioli, Mariana Bonilla, Vadim N. Gladyshev, Mariana Boiani, Gustavo Salinas

**Affiliations:** 1 Cátedra de Inmunología, Facultad de Química and Facultad de Ciencias, Universidad de la República, Montevideo, Uruguay; 2 Grupo de Química Medicinal, Laboratorio de Química Orgánica, Facultad de Química and Facultad de Ciencias, Universidad de la República, Montevideo, Uruguay; 3 Unidad de Biología Parasitaria, Facultad de Ciencias, Universidad de la República, Montevideo, Uruguay; 4 Division of Genetics, Department of Medicine, Brigham and Women's Hospital and Harvard Medical School, Boston, Massachusetts, United States of America; Instituto de Biociencias - Universidade de São Paulo, Brazil

## Abstract

Parasitic flatworms are responsible for serious infectious diseases that affect humans as well as livestock animals in vast regions of the world. Yet, the drug armamentarium available for treatment of these infections is limited: praziquantel is the single drug currently available for 200 million people infected with *Schistosoma* spp. and there is justified concern about emergence of drug resistance. Thioredoxin glutathione reductase (TGR) is an essential core enzyme for redox homeostasis in flatworm parasites. In this work, we searched for flatworm TGR inhibitors testing compounds belonging to various families known to inhibit thioredoxin reductase or TGR and also additional electrophilic compounds. Several furoxans and one thiadiazole potently inhibited TGRs from both classes of parasitic flatworms: cestoda (tapeworms) and trematoda (flukes), while several benzofuroxans and a quinoxaline moderately inhibited TGRs. Remarkably, five active compounds from diverse families possessed a phenylsulfonyl group, strongly suggesting that this moiety is a new pharmacophore. The most active inhibitors were further characterized and displayed slow and nearly irreversible binding to TGR. These compounds efficiently killed *Echinococcus granulosus* larval worms and *Fasciola hepatica* newly excysted juveniles *in vitro* at a 20 µM concentration. Our results support the concept that the redox metabolism of flatworm parasites is precarious and particularly susceptible to destabilization, show that furoxans can be used to target both flukes and tapeworms, and identified phenylsulfonyl as a new drug-hit moiety for both classes of flatworm parasites.

## Introduction

Flatworm infections are a major cause of human disability and mortality in many developing countries, and remains as one of the most important challenges for medicine in the 21^st^ century [1], [2]. In addition, many flatworms parasitize livestock and cause economically important diseases. Flatworm parasites include two major lineages: flukes (class Trematoda) and tapeworms (class Cestoda). Liver fluke disease is caused by endoparasitic trematodes of the genus *Fasciola*. *Fasciola hepatica*, the common liver fluke, widely distributed in temperate climates, causes massive economic losses to livestock production due to reduction in meat, wool and milk output in infected animals. Its significance as an emerging food-borne zoonosis in parts of Latin America and Africa, with millions at risk of infection, has been recognized by the WHO [3]. Fasciolosis control is dependent on repeated treatment with anthelmintic drugs. However, resistant strains against triclabendazole, the drug of choice, have appeared in Europe and Australia [4]. Cystic echinococcosis or cystic hydatid disease caused by the larval stage of the dog tapeworm *Echinococcus granulosus*, the most widespread zoonosis caused by a cestode, remains a serious threat to human health. Control programs of cystic echinococcosis are based on repeated anthelmintic treatment of dogs with praziquantel [5]. For the larval stage, chemotherapy with benzimidazoles is combined with surgical removal of the cyst. In the case of alveolar echinococcosis or alveolar hydatid disease, caused by *Echinococcus multilocularis* infection, continuous chemoprophylaxis with benzimidazoles leads to a good quality of life for most patients with the chronic disease [6]. Despite the medical relevance of flatworm infections, the tools available to their control are very limited: there is no single vaccine available for a human flatworm infection, and the pharmacological arsenal for many of them consists of just a single drug, for which there is concern of drug resistance emergence and/or spreading [7], [8]. Indeed, praziquantel is the single effective drug for schistosomiasis treatment, the main chronic disease caused by flatworms, infecting 200 million people in tropical regions. Despite the urgent need for novel effective anti-flatworms drugs, discovery and development research has been sparse over the last decade. A rational target-based approach to the discovery of drug candidates holds promise to accelerate the process.

An unusual metabolic aspect of flatworm parasites is their unique array of thiol-based redox pathways. In contrast to most organisms, including their mammalian hosts, flatworm parasites possess the selenoenzyme thioredoxin glutathione reductase (TGR) as a single core enzyme for thioredoxin- and glutathione-dependent pathways [9], [10], [11]. Thus, antioxidant defenses, redox homeostasis and DNA synthesis in flatworm parasites depends on a single essential enzyme that has been validated as a drug target for *Schistosoma mansoni* infection. This work led to high throughput screening of TGR inhibitors and to the identification of oxadiazoles, among others, as new drug leads for the control of schistosomiasis [12], [13], [14]. It has also recently been demonstrated that auranofin, a specific gold inhibitor of selenocysteine (Sec) containing TRs and TGRs, kills *in vitro Echinococcus granulosus* and *Taenia cracisseps* larval worms, indicating that TGR is an essential enzyme in cestodes [15], [16]. Tapeworm TGR also fulfills other requirements as a drug target: it is constitutively expressed, there is a low cost and simple biochemical assay to test its activities, and importantly it is a “druggable enzyme”. The Sec residue in TGRs contains a nucleophilic, highly reactive side chain that is a highly susceptible target site for electrophiles. Based on these premises, we selected 65 compounds as candidate TGR inhibitors from our chemical library of compounds belonging to different families of electrophililic systems as well as known TR and TGR inhibitors. We identified new oxadiazole *N*-oxides (also known as furoxans), a quinoxaline, and a thiadiazole as inhibitors for flukes and tapeworms. Furthermore, several active compounds belonging to the different families contain the phenylsulfonyl moiety suggesting that this group is a potential new pharmacophore to target flatworm TGRs. The identified inhibitors of TGR were able to kill *in vitro* cestode larval worms of *E. granulosus* and the invasive juvenile stage of *F. hepatica in vitro*.

## Materials and Methods

### Selection of compounds

**Figure 1:**
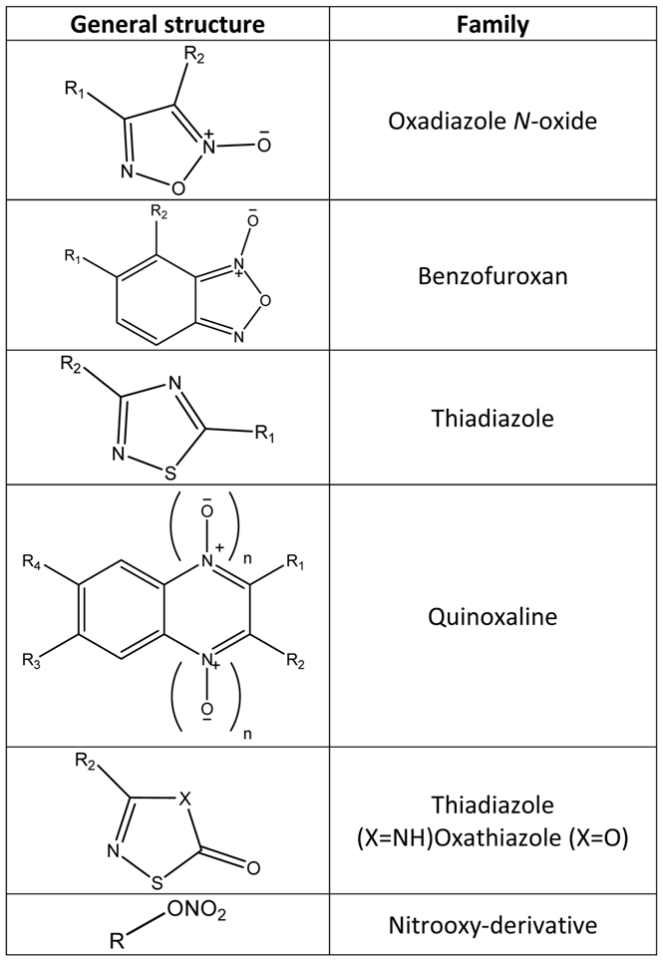
Structures of the families of compounds assayed as inhibitors of flatworm TGR (all compounds assayed are shown in Table S1). R, R_1_-R_4_: variable.

Compounds were selected from previously synthesized products available from our chemical library (see references in **Table S1**), taking into account previously identified *S. mansoni* TGR inhibitors [13], [14], *Plasmodium falciparum* TR inhibitors [17], and additional compounds with electrophilic groups. In total, 65 compounds belonging to the following structural families were selected: oxadiazole *N*-oxide or furoxan (21), benzofuroxan (28), thiadiazole (11), quinoxaline (3), nitrooxy-derivative (1) and oxathiazole (1). **Figure 1** shows the general structures of the compound families assayed as inhibitors of TGR; **Table S1** shows the structures of every compound assayed.

### Cloning, expression and purification of *E. granulosus* and *F. hepatica* wild-type TGRs

The construct for recombinant expression of *E. granulosus* TGR was previously generated [15].The construct for *F. hepatica* TGR was generated using the same methodology [18]. Both TGR constructs contained the Sec insertion sequence (SECIS) element of *E. coli* formate dehydrogenase H at a 10 nucleotide distance from the penultimate UGA_Sec_ codon, to allow stop codon recoding to Sec, as previously described [15], [18]. For recombinant protein expression, TGR constructs were used to transform *E. coli* BL21(DE3) cells previously transformed with pSUABC, a plasmid that encodes *selA*, *selB* and *selC*, supporting high level expression of the genes involved in Sec incorporation [19]. Expression of TGRs was carried out following the protocol described in [20], which has been optimized for expression of selenoproteins. Essentially, induction of recombinant protein was carried out with 100 µM IPTG in late exponential phase (OD_600_ = 2.4), during 24 h at 24°C. Recombinant clones were grown in modified LB media according to [21], supplemented with 0.1 g/L cysteine and 0.37 g/L methionine [22], in the presence of kanamycin (50 µg/mL), and chloramphenicol (33 µg/mL). At the time of induction the culture was supplemented with 5 µM sodium selenite, 20 µg/mL riboflavin, 20 µg/mL pyridoxine and 20 µg/mL niacin according to [21]. The bacterial cultures were centrifuged and the pellets resuspended in modified Ni-NTA lysis buffer (300 mM NaCl, 50 mM sodium phosphate, 20 mM imidazole, pH 7.2) containing 1 mM PMSF and 1 mg/mL lysozyme, and sonicated (10 pulses of 1 min with 1 min pauses). The lysates were centrifuged for 1 h at 30,000 g, and supernatants applied to a Ni-NTA column (Qiagen), washed with 300 mM NaCl, 50 mM sodium phosphate, 30 mM imidazole, pH 7.2, and eluted with 250 mM imidazole. The protein-containing fractions were applied to PD10 desalting columns (GE Healthcare) using phosphate-buffered saline (PBS), 150 mM potassium chloride, 50 mM sodium phosphate, pH 7.2. Fractions containing the recombinant protein were stored at −70°C before use. Total protein concentration and FAD content were determined spectrophotometrically at 280 (ε = 54.24 mM^−1^ cm^−1^) and 460 nm (ε = 11.3 mM^−1^ cm^−1^), respectively. The selenium content of selenoproteins was determined by atomic absorption using a Plasma Emission Spectrometer (Jarrell-Ash 965 ICP) in Chemical Analysis Laboratory, University of Georgia. Active selenoenzyme concentrations were calculated considering their selenium contents. The purity of recombinant proteins was analyzed by running 10% SDS-PAGE gels, under reducing conditions, and by size exclusion chromatography on a Superose 12 column (GE Healthcare). Fractions containing recombinant proteins were stored at −70°C prior to use.

### DTNB reduction assay for TR activity

The reduction of 5,5′-dithiobis(2-dinitrobenzoic acid) (DTNB) with concomitant NADPH oxidation was determined by the increase in absorbance at 412 nm due to the formation of 5′-thionitrobenzoic acid (TNB) (ε = 13600 M^−1^ cm^−1^) [23]. The reaction mixtures contained 100 µM NADPH and 5 mM DTNB.

### GSSG reduction assay

The GR activity was assayed as the NADPH-dependent reduction of oxidized glutathione (GSSG), which is followed by the decrease in absorbance at 340 nm due to NADPH oxidation (ε = 6200 M^−1^ cm^−1^) [24]. The reaction mixtures contained 100 µM NADPH and 100 µM GSSG to avoid conditions of hysteresis [15], [25], [26], [27].

### Inhibition studies

Inhibitor stock solutions were prepared at a final concentration of 10 mM or 100 mM in dimethylsulfoxide (DMSO). The screening was performed using the DTNB assay for TR activity. *E. granulosus* recombinant TGR was used at a 1 nM final concentration in all assays. For the initial screening compounds were assayed at a 10 µM final concentration, except for a few cases in which compounds were tested at 1 µM, due to non-constant baselines at 10 µM in control experiments without enzyme. In all cases TGR was preincubated during 3 minutes with NADPH and compounds to test, and the reaction started by addition of DTNB and followed for 3 minutes. All assays were performed in duplicate. In every case, a control progress curve without enzyme was performed to control for non-catalyzed reactions between substrates and inhibitors. The percentage of TR inhibition was calculated as follows: % Inhibition = 100−(v_i_/v_o_)×100, where v_i_ and v_o_ correspond to the initial velocities of TNB formation (µM)/t (s) with and without inhibitor, respectively. Compounds inhibiting more than 30% the TR activity under the conditions assayed were used in further experiments. All active compounds were assayed for inhibition of GR activity using recombinant *E. granulosus* TGR and also screened for TR and GR inhibition of *F. hepatica* TGR. Additional characterization was performed for compounds that showed more than 50% inhibition under the conditions assayed. We evaluated whether inhibitors were slow-binding using preincubation times ranging from 3 to 120 minutes. In addition, in order to study the binding of inhibitors NADPH-enzyme-inhibitors mixes were preincubated as described above during 30 minutes and then the mixes were applied to a PD10 column, in order to remove the excess of unbound inhibitor. The TR activity of the eluates was evaluated by adding NADPH and DTNB. Control experiments without inhibitors were carried out in parallel.

### *In vitro* culture of larval worms

50,000 protoscoleces, obtained from asceptical punction of a single hydatid cyst from bovine lung, were washed several times with PBS and then incubated at 37°C, 5% CO_2_, in DMEM supplemented with antibiotics and 20 mM HEPES, pH 6.8. In all cases 1,000 protoscoleces were treated with 100, 50 or 20 µM inhibitor concentrations. Protoscoleces were observed under the microscope every 4 h and endpoint viability assessed by exclusion of the vital dye eosin [28] at 48 h. The infected bovine viscera was obtained as part of the normal discard processing of the abattoir.

### *In vitro* culture of newly excysted juvenile

Metacercariae of *F. hepatica* were purchased from Baldwin Aquatics Inc. (Monmouth, Oregon). The *in vitro* excystment was performed as previously described in [29], with minor modifications. Briefly, metacercariae were placed in a 100 mm filter and incubated 3 min at room temperature with 1% sodium hypochlorite to remove contaminants. After an exhaustive wash in double distilled H_2_O, the metacercariae were incubated at 39°C in activation media (25 mM HCl and 16,5 mM *l*-cysteine, 0.1% sodium taurocholate, 60 mM NaHCO_3_, 70 mM NaCl pH 8.0), and the excystment process was monitored under the microscope. After 90–180 min of incubation, newly excysted juveniles (NEJ) began to emerge, and were collected, washed several times with RPMI-1640, pH 7.2, and transferred into 6 wells plates and kept in culture at 37°C under 5% CO_2_ with RPMI-1640 media containing 200 U/mL Penicillin G sulfate, 500 ng/mL amphotericin B, 10 mM HEPES. In all cases 250 NEJ were treated with 20 µM concentration of inhibitors. NEJ were observed under the microscope every 4 h and endpoint viability assessed by exclusion of the vital dye eosin at 48 h.

### Structure-activity studies

Three types of descriptors were analyzed in this study: constitutional descriptors, molecular properties and topological charge indices. The program E-DRAGON (www.vcclab.org/lab/edragon/) was used to generate the descriptors using the SMILES code of each molecule. The initial number of descriptors was submitted to the following reduction procedure: i) descriptors with constant values for 80% or more of the molecules were excluded; ii) pairwise correlation was done, and a descriptor was eliminated if the correlation coefficient with another descriptor was equal to or higher than 0.9; and iii) only descriptors able to discriminate between activity classes were selected (t test, *t*-value>2). For this purpose, compounds were classified as actives (TGR inhibition >50%) and inactives (TGR inhibition <50%). All the reduced descriptors were autoscaled using the mean and standard deviation. The ability of the descriptors to discriminate between activity classes was analyzed using principal components analysis [30]. The best combination of descriptors was used to classify compounds using hierarchical cluster analysis (HCA).

### Molecular modeling

Molecular modeling of compounds was carried out using the Spartan'04 program package [31]. Molecular mechanics (MMFF) was used for preliminary structure optimization and conformational search as implemented in the program. The minimum energy conformer was further optimized using density functional theory (B3LYP/6-31G*). At this level the following properties were calculated: highest occupied molecular orbital (HOMO) energy (EHOMO), lowest unoccupied molecular orbital (LUMO) energy (ELUMO), HOMO-LUMO energy gap (GAP), octanol/water partition coefficient using Ghose-Crippen method (LogP) [32], solvation energy using Truhlar model SM5.4 (Esolv) [33], and module of dipolar moment (μ).

### Compounds toxicity assays

Murine fibroblasts (L929 cell line) were incubated at 37° with compounds 1, 2, 3, 5, 22 and 61 (range of concentrations 5–160 µM) in DMEM supplemented with glutamine and 5% heat-inactivated fetal bovine serum. Viability was examined 48 hours later using the MTT (3-(4,5-Dimethylthiazol-2-yl)-2,5-diphenyltetrazolium bromide) reduction assay [34]. Citotoxicity profiles for the other active compounds were previously published (see Toxicity Profile in **Text S1**, and references therein). Additional toxicity assays were performed for those compounds that showed more than 50% inhibition under the initial screening conditions and effectively killed *E. granulosus* and *F. hepatica* at a 20 µM concentration (compounds 1, 2, 3 and 50). These assays were carried out using human primary cell cultures and monitoring toxicity for a longer time (96 hours). Peripheral blood mononuclear cells (PBMC, mostly consisting of lymphocytes) were isolated from whole human blood by centrifugation through Ficoll-Hypaque solution (Histopaque® 1077, SIGMA-ALDRICH), according to Procedure N° 1077 from the manufacturers. Cells were incubated in RPMI 1640 supplemented with glutamine, 10% heat-inactivated fetal bovine serum and antibiotics at concentrations of 10, 20, 40 and 80 µM of each compound. Assays were carried out in 96 well culture plates containing 150.000 cells per well. Viability was examined after 96 hours of culture as described above and by exclusion of the vital dye trypan blue. A written consent was obtained from the donor according to regulation N° 282 of the Ethic Committee of the Faculty of Chemistry (Universidad de la República) and the National Decree N° 379/008.

## Results

### The identified TGR inhibitors belong to different families of compounds

**Figure 2:**
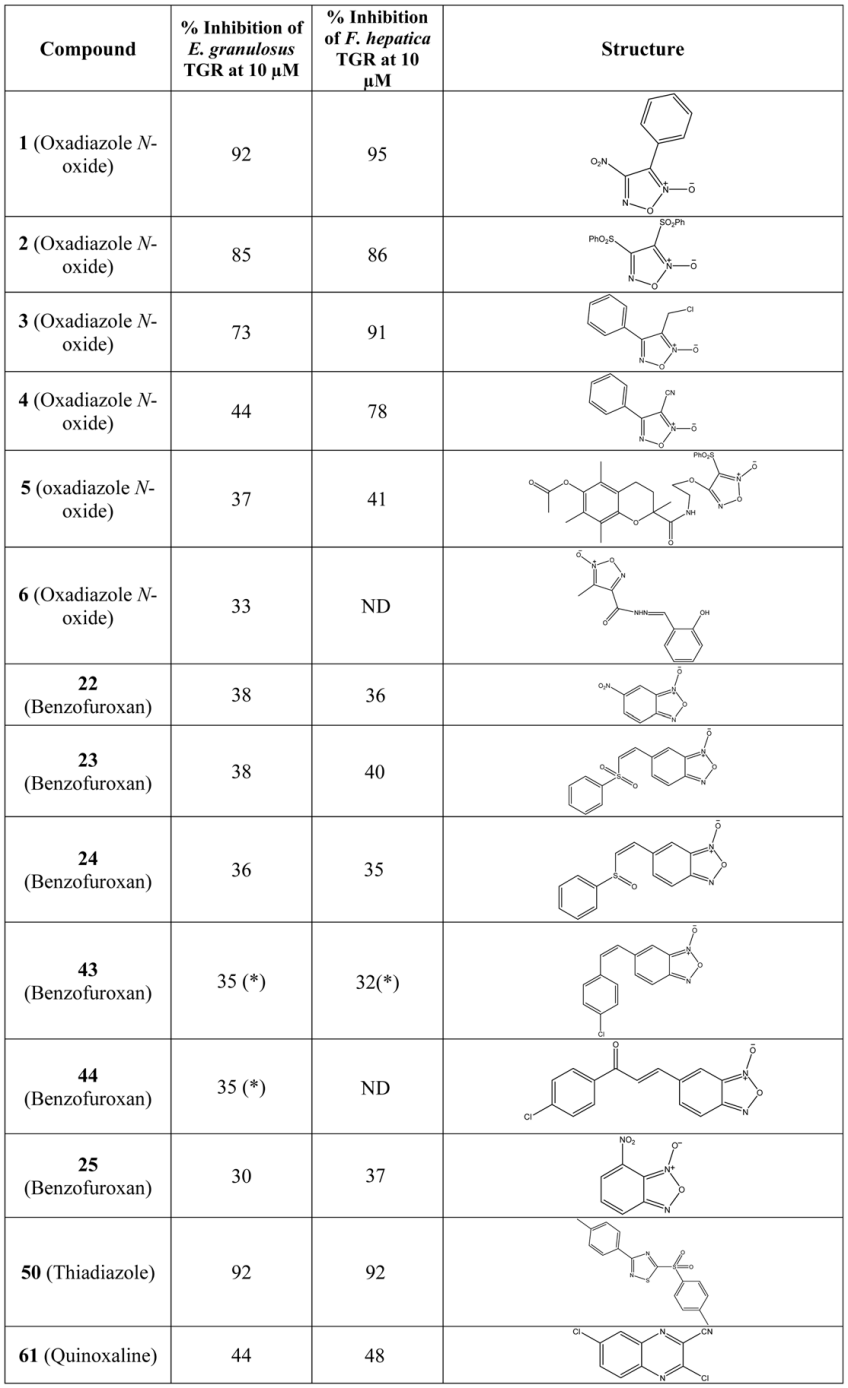
Compounds that inhibit TR activity of flatworm TGRs. The % of inhibition of *E. granulosus* and *Fasciola hepatica* TGR and molecular structures are shown for each of the compounds tested. The screening was performed at 10 µM inhibitor concentration, except in those cases marked with an asterisk, which were assayed at 1 µM (at 10 µM control experiments for these compounds without enzyme showed non-constant base lines). ND: not determined since no larvicidal effect was observed at the highest concentration for *E. granulosus*.

The sixty five compounds selected as putative TGR inhibitors are shown in **Table S1**. These compounds belong to 6 different families, which are depicted in **Figure 1**. In total, we examined 21 oxadiazole *N*-oxides (also known as furoxans), 26 benzofuroxans (these compounds represent a particular type of oxadiazole *N*-oxides), 11 thiadiazoles, 3 quinoxalines, 1 oxathiazole and 1 nitrooxy-derivative. The furoxan family included the compound **4** (4-phenyl-1,2,5-oxadiazole-3-carbonitrile-2-oxide, see **Table S1**), a well-known *S. mansoni* TGR inhibitor with capability to release nitric oxide (NO) [12], [35]. An initial screening for inhibitors was carried out using the TR assay with DTNB as a substrate. The rationale to screen the compounds using the TR assay was that both thioredoxin and glutathione reductase activities are dependent on the TR module of TGR, and therefore its inhibition leads to ablation of all thiol-disulfide oxidoreductase activities of TGR. Initially, the compounds were tested at 10 µM concentration; in a few instances inhibitors were tested at 1 µM (see Material and Methods). Several oxadiazole *N*-oxides, benzofuroxans, one quinoxaline and one thiadiazole significantly inhibited TR activity. The results are shown in **Figures 2** **and** **3**. For further work, we selected those compounds that inhibited TR activity more than 30% when assayed at either 1 or 10 µM concentration. Under these criteria, 14 of the 65 tested compounds were shown to inhibit the TR activity of TGR.

**Figure 3:**
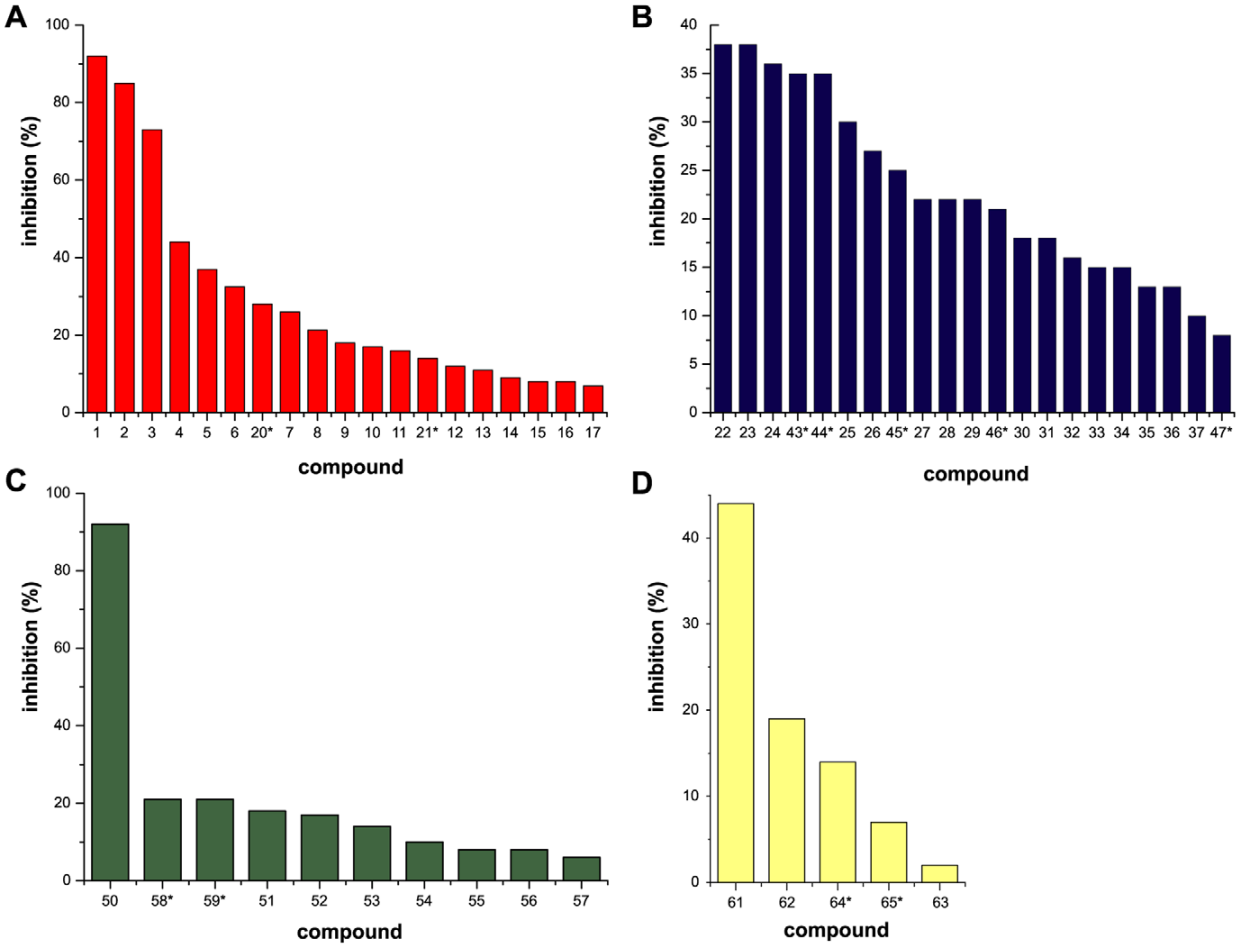
Screening of TGR inhibitors. The % of inhibition of TR activity of *E. granulosus* TGR for each compound is shown (compound structures can be found in **Table S1**). The screening was carried out with 10 µM inhibitors, except for those marked with an asterisk, which were assayed at 1 µM (these compounds showed non-constant baselines at 10 µM in control experiments without enzyme). A 1 nM TGR was used in all cases. **a.** Oxadiazole *N*-oxides, **b.** Benzofuroxans, **c.** Thiadiazoles, **d.** Various compound families: quinoxalines (**61**, **62** and **65**), nitrooxy (**64**) and oxathiazole (**63**).

**Figure 4:**
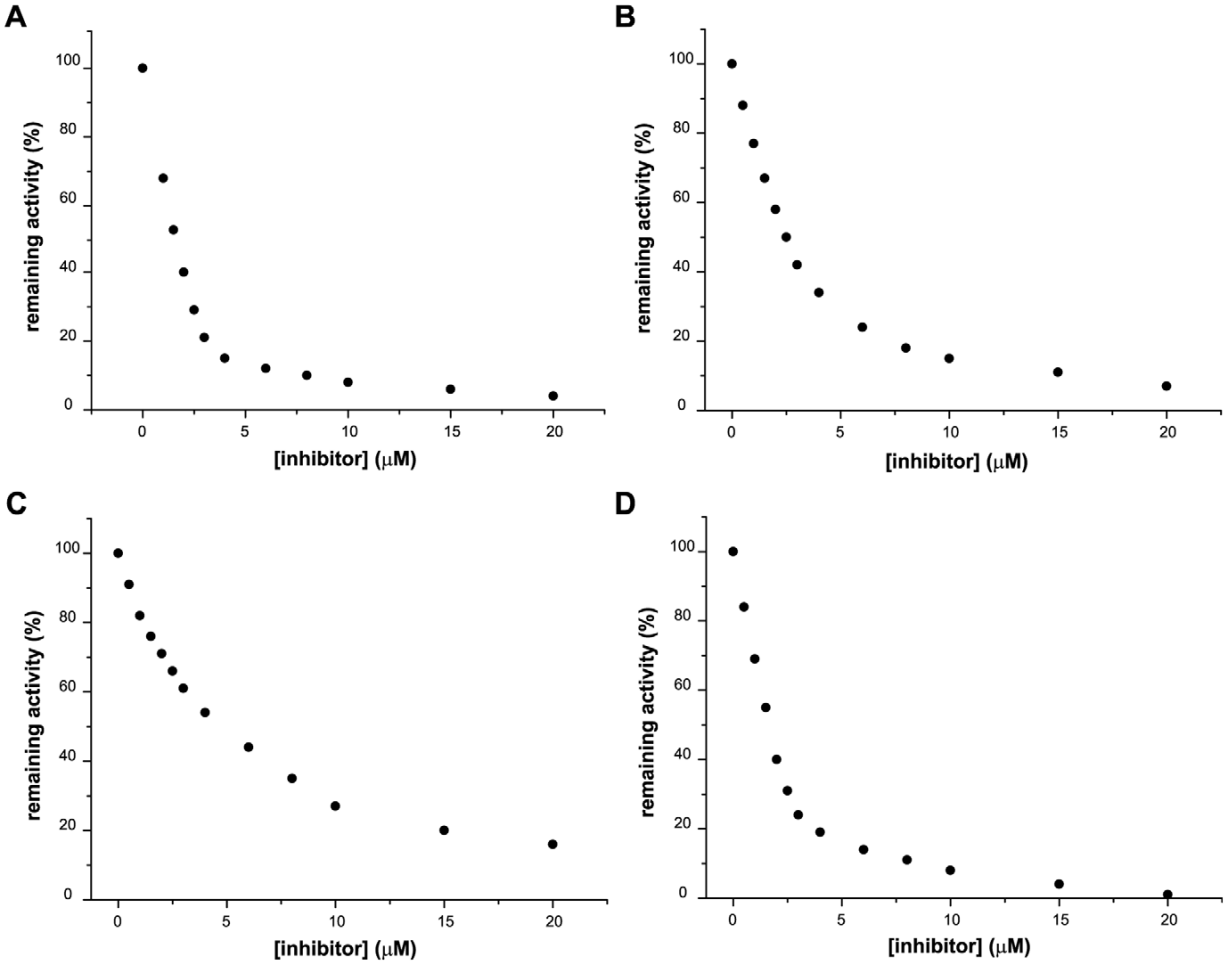
Inhibition of TR activity of TGR by active compounds. Remaining TR activity as a function of inhibitor concentration is plotted for **a. 1**, **b. 2**, **c. 3** and **d. 50**. 1 nM TGR was used in all assays.

A major finding was that drug hits for *S. mansoni* TGR (oxadiazole *N*-oxides and quinoxalines) also affected the activity of *E. granulosus* TGR: several furoxans and benzofuroxans and one of three quinoxalines inhibited this cestode TGR. The other major finding was that one thiadiazole was a very good inhibitor of *E. granulosus* TGR; indeed, TR inhibition by this thiadiazole was comparable to the inhibition achieved by the most efective oxadiazole *N*-oxides. The most active TGR inhibitors (*i.e.* those showing more than 50% inhibition in the screening) were assayed over a wide range of concentrations of active compounds (**Figure 4**). It is interesting to note that the reference compound that inhibited *S. mansoni* TGR (compound **4**) [14] also inhibited *E. granulosus* TGR. For the TR inhibitors identified, we then assayed the inhibition of GR activity of TGR. All of them inhibited GR activity; this was expected since inhibition of the TR module leads to inhibition of both TR and GR activities.

### The identified tapeworm TGR inhibitors are also active against fluke TGR

The identified *E. granulosus* TGR inhibitors were assayed for inhibition of TR and GR activities of *F. hepatica* TGR. All *E. granulosus* TGR inhibitors were also active against recombinant *F. hepatica* TGR (see **Figure 2**). The percentage of inhibition of TR activities of *F. hepatica* and *E. granulosus* TGRs were similar. The oxadiazoles **3** and **4** showed higher percentage of inhibition of TR activity of *F. hepatica* TGR (91% and 78% respectively) than the corresponding inhibition percentage of *E. granulosus* TGR (73% and 44%, respectively) at 10 µM. Most remarkably, the thiadiazole identified as the best *E. granulosus* TGR inhibitor (compound **50**) also inhibited *F. hepatica* TGR; inhibition of TR activity at10 µM was 80% (92% in the case of *E. granulosus* TGR).

### The identified TGR inhibitors are slow-binding and nearly irreversible

In order to further characterize the best inhibitors we examined whether the extent of inhibition was time dependent. This cannot be achieved by following a single time course experiment since initial velocity conditions, *i.e.* constant velocities, are lost early during the DTNB assay At constant inhibitor concentration, longer preincubation times led to higher inhibition for the all compounds, indicating that these molecules are slow-binding inhibitors. The results for compound 1 are shown in **Table 1**; similar results were obtained for compounds 2, 3 and 50. Indeed, the concentrations needed to achieve 50% inhibition after 120 min preincubation time was 10-fold lower than those needed after 3 min preincubation (data not shown). These results suggested that these inhibitors could be irreversible. To study the reversibility of enzyme-inhibitor binding we preincubated NADPH-enzyme-inhibitors mixes in reaction buffer during 30 minutes and then applied the mixes to a PD10 column in order to remove the excess of unbound inhibitor. For the four compounds inhibition persisted after removal of the excess of inhibitors. In other words, no TR activity was recovered in the eluates. Furthermore, no activity was recovered even after allowing for a 30 minutes equilibration of the eluates (*i.e.* allowing for inhibitor-enzyme desorption). These results indicated that the inhibitors are virtually irreversibly bound to the enzyme (*i.e.* either covalently or tightly bound to the enzyme).

**Table 1:** Effect of preincubation time on TR activity inhibition of TGR.

Preincubation time (min)	Inhibition % (*)
**3**	48
**30**	82
**60**	92
**90**	96
**120**	100

(*) 1.5 µM of compound 1 was incubated with 1 nM enzyme in the presence of NADPH and the reaction started by the addition of DTNB. Control experiments without inhibitors were carried out in parallel to determine the corresponding v_o_ (initial velocities without inhibitors).

### Preliminary structure-activity relationships

The indication of a SAR pattern is important to identify hit-to-lead and lead-to-drug transitions [36]. Herein, three types of descriptors were used to establish a structure-TGR inhibition relationship; constitutionals descriptors, molecular properties, and topological charge indices. Initially, we used principal components analysis (PCA) to assess descriptors that may explain the TGR inhibitory activity. For this, compounds were separated in activity classes using our internal definition of hit compound (TGR inhibition >50%). A low activity class was considered including compounds with TGR inhibition in the range 30–50%. From this analysis, the following descriptors were found related to TGR inhibition: molecular volume, number of rings and of six-membered rings, charge distribution, topological polar surface area, and unsaturation index. Overall, active compounds were small in the group of tested compounds. All four active compounds had an aromatic ring attached to heterocycle (**1** and **3**) or in its proximity (**2** and **50**). The presence of the aromatic ring seems to be essential for TGR activity since methylfuroxans (**6**, **8–19**, and **21**, **Table S1**) were inactive, with the exception of compound **6** that was moderately active.

Using the descriptors selected by PCA, we then applied hierarchical cluster analysis (HCA) to obtain an unbiased analysis of molecular properties related to enzyme inhibition. Here, compounds were clustered according to structure similarity without any previous activity tag. HCA identified three clusters. The first cluster included only inactive compounds, methylfuroxans and phenoxybenzofuroxans. These compounds displayed low electrophilicity among their family classes, which could explain their lack of activity. The second cluster included the active furoxan **2** and the active thiadiazole **50**, along with benzofuroxans **23** and **24** that had low activity. All these compounds had an arylsulfonyl (or arylsulfinyl) moiety that could act as an electron-withdrawing group increasing the heterocycle reactivity or could be itself a pharmacophore. It is worth noting that the spatial distribution of the phenylsulfonyl (or phenylsulfinyl) moiety is relevant for activity. For instance, compounds **23** and **24**, both *cis* isomers, displayed low activity at 1 µM and the corresponding *trans* isomers (**42** and **40**, **Table S1**) were inactive. A similar spatial requirement was observed for compound **43**, the only ethenylbenzofuroxan that displayed low activity at 1 µM, the corresponding *trans* isomer is inactive. This effect could hardly be ascribed to differences in electrophilicity. In fact molecular modeling showed no differences in electronic properties for derivatives **24** and **40**, suggesting that these substituents could be participating in an interaction with TGR. Finally, the third cluster included active compounds **1** and **3** along with low activity compounds **4** (furoxan derivative), **22** and **25** (benzofuroxan derivatives), and quinoxaline **61**. The majority of these compounds have the presence of an electrophilic moiety such as chloromethyl, nitroimine, or nitro in common.

Remarkably, the active furoxan derivative **2** was clearly separated from other furoxan derivatives, and was clustered with the active thiadiazole **50**. Both compounds had a phenylsulfonyl substituent (*p*-methylphenylsulfonyl in **50**) attached to the heterocycle. Altogether, this finding suggested that the phenylsulfonyl moiety is a new pharmacophore, while the heterocycle ring was acting as a scaffold. While this hypothesis requires additional studies, it also supports high structural diversity and flexibility in the design of TGR inhibitors. On the other hand, the fact that active compounds were separated into two groups, suggests the occurrence of different mechanisms of enzyme inhibition.

**Figure 5:**
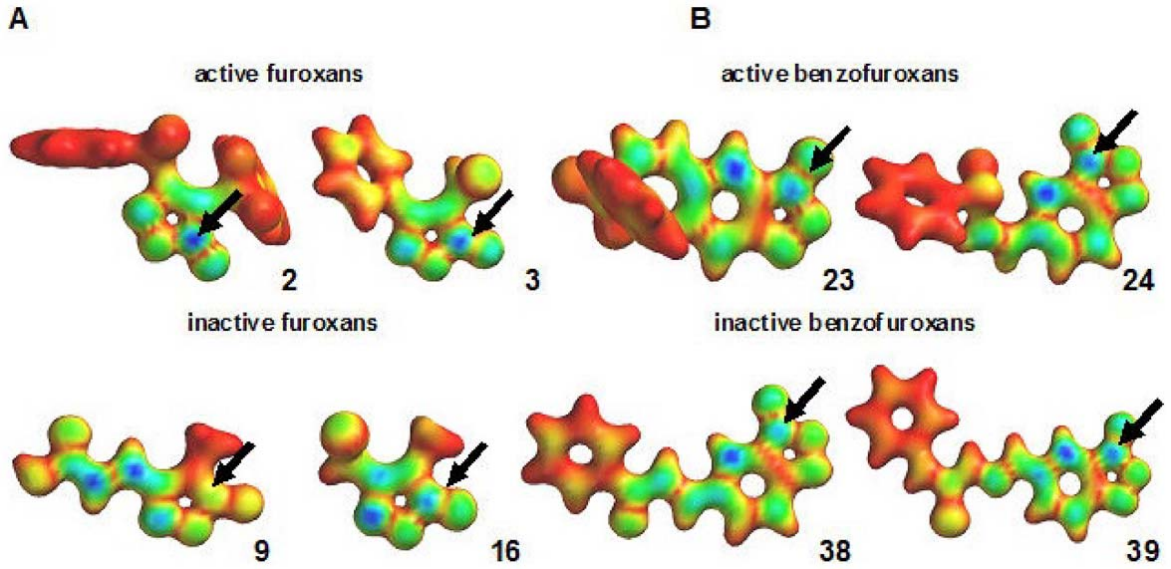
LUMO maps allow identification of electrophilic centers. LUMO mapped onto a bond surface for studied compounds: a. furoxans, b. benzofuroxans. Blue areas correspond to regions in which the molecule is most susceptible to nucleophilic attack in active compounds the nitrogen of *N*-oxide moieties. The arrows show the difference on LUMO contribution of these nitrogens between active and inactive compounds of the same family.

To further study molecular properties related to TGR inhibition we used molecular modeling to determine the electronic structure of a series of furoxans and benzofuroxans (**Table 2**). Overall, the results obtained supported the PCA/HCA analysis. The energies of frontier orbitals (highest occupied molecular orbital or HOMO and lowest unoccupied molecular orbital or LUMO) were similar for active and inactive compounds in both families. The energy of the frontier orbitals is related to the compound's reactivity, implying that active and inactive compounds have similar reactivity, and differences in TGR inhibition are probably related to other factors. While HOMO and LUMO energies are global properties, related with the reactivity of the whole molecule, their values contain no information on which atom of the molecule reacts. To assess local reactivity we used molecular orbital maps (**Figure 5**). For furoxans, the LUMO maps showed that the nitrogen of the *N*-oxide moiety is the main contributor in active derivatives **2** and **3** (**Figure 5A**). In contrast, for inactive derivatives **9** and **16** the main contributor is nitrogen 3 in the heterocycle. A similar pattern was observed for benzofuroxans (**Figure 5B**). Among the other global properties calculated the only one that was related to TGR inhibition was lipophilicity (LogP). Active furoxans are more hydrophobic (higher LogP) than inactive ones, mainly due to the presence of aromatic rings and the absence of hydrophilic substituents such as OH, COOH, and NH_2_. This suggests interactions with hydrophobic residues in the enzyme are important for the biological activity.

**Table 2:** Characteristics of active and inactive compounds.

Family	Compound	EHOMO^a^ (eV)	ELUMO^b^ (eV)	GAP^c^ (eV)	LogP^d^	Esol^e^ (kcal/mol)	μ^f^ (D)
**Active furoxans**	**1**	−6.94	−3.21	−3.73	3.9	3.11	3.66
	**2**	−7.46	−2.49	−4.97	4.42	−12.75	6.77
	**3**	−6.88	−1.95	−4.93	4.54	−1.73	4.73
**Inactive furoxans**	**7**	−7.09	−2.11	−4.98	3.16	−8.26	4.36
	**9**	−6.75	−2.60	−4.15	3.17	−5.16	2.92
	**11**	−7.04	−2.64	−4.40	2.39	−1.72	1.58
	**16**	−6.81	−1.73	−5.08	3.4	−2.44	3.61
**Active benzofuroxans**	**23**	−6.35	−3.03	−3.32	5.05	−10.71	3.95
	**24**	−6.1	−2.74	−3.36	5.12	−10.70	7.64
**Inactive benzofuroxans**	**34**	−5.95	−3.11	−2.84	3.76	−14.64	7.16
	**38**	−6.19	−3.09	−3.10	5.40	−3.67	2.81
	**39**	−5.86	−2.80	−3.06	5.37	−9.56	7.01
	**40**	−6.07	−2.85	−3.22	5.00	−10.77	3.07

a Highest occupied molecular orbital energy.

b Lowest unoccupied molecular orbital energy.

c HOMO-LUMO energy gap.

d Octanol/water partition coefficient using Ghose-Crippen method.

e Solvation energy using Truhlar model SM5.4.

f Dipolar moment.

### Identified inhibitors killed *E. granulosus* larval worms *in vitro*

**Figure 6:**
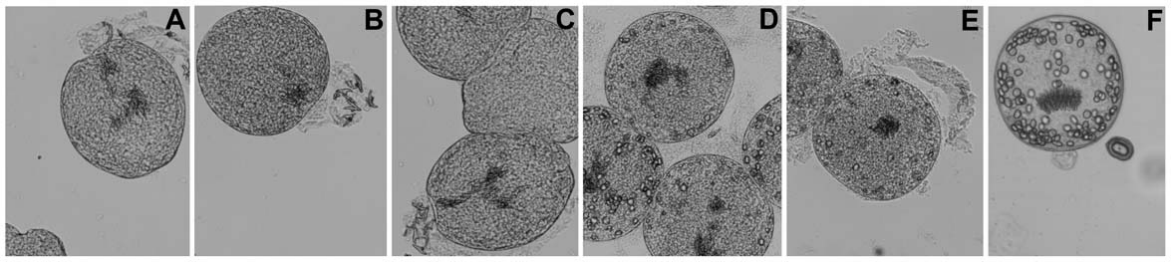
Effect of TGR inhibitors on *E. granulosus* protoscoleces, tested at 20 µM during 48 h. a. **2** (oxadiazole *N*-oxide), b. **1** (oxadiazole *N*-oxide), c. **4** (oxadiazole *N*-oxide), d. **61** (quinoxaline), e. **50** (thiadiazole), f. control.

To test whether *E. granulosus* TGR inhibitors were active against parasites, we assessed the effect of active compounds against *E. granulosus* larval worms (protoscoleces) *in vitro*. As a control for *in vitro* studies, we used an oxadiazole *N*-oxide, **19**, and a benzofuroxan, **42**, which did not display any TGR inhibition. The results are shown in **Table 3** and indicate that there is a very good correlation between TGR inhibition and *in vitro* killing of larval worms. The fact that inhibitors of TGRs killed larval worms, but the control oxadiazole *N*-oxide and benzofuroxan did not is a strong indication that lethality is due to inhibition of TGR. At the concentration of 100 µM, 11 out of the 14 compounds assayed killed 100% of larval worms (**Table 3**), one of them showed a moderate larvicidal effect (**22**) and only 2 of the 14 compounds assayed (**44** and **6**) did not exhibit any larvicidal activity within 48 h. At 50 µM most inhibitors also killed larval worms, but the kinetics of killing differed and it was faster for most active compounds (data not shown). The best TGR inhibitors (the three oxadiazole *N*-oxides **1**, **2**, and **3**, and the thiadiazole **50**) were the most active compounds: they killed between 30 and 40% of the protoscoleces at 20 µM, after 48 h of culture (see **Table 3**). Interestingly, compound **4** that was not among the best inhibitors of TR activity, exhibited a larvicidal effect comparable to the best inhibitors. Similarly, in *S. mansoni* this compound was one of the most active against the parasite but not the best TGR inhibitor [35]. Most benzofuroxans that were moderately active inhibitors of TGR were also moderately effective in killing *E. granulosus* protoscoleces. **Figure 6** shows protoscoleces treated with inhibitors and control compounds. The effect of compounds resulted in total disintegration of *E. granulosus* protoscolex parenchyma.

**Table 3:** *E. granulosus* protoscolex viability *in vitro* after 48 h incubation with TGR inhibitors at different concentrations.

Compound	Protoscolex viability (%) at 100 µM	Protoscolex viability (%) at 50 µM	Protoscolex viability (%) at 20 µM	% TR inhibition at 10 µM
**2**	0	0	60	85
**1**	0	0	63	92
**3**	0	0	70	73
**4**	0	0	70	44
**5**	0	0	100	37
**6**	100	ND	ND	38
**43**	0	0	90	35(*)
**25**	0	0	90	30
**23**	0	0	90	38
**24**	0	0	100	36
**22**	30	65	100	38
**44**	100	ND	ND	35(*)
**50**	0	0	65	92
**61**	0	0	90	44
**19**	100 (control)	ND	ND	6
**42**	100 (control)	ND	ND	0

ND: not determined (since no effect was observed at the highest concentration). **42** (benzofuroxan control) and **19** (oxadiazole *N*-oxide control). A control with vehicle alone (DMSO) was also included and did not affect protoscolex viability.

(*) % of inhibition of TR activity at 1 µM not at 10 µM.

### Identified inhibitors killed *F. hepatica* newly excysted juvenile larval worms *in vitro*

**Figure 7:**
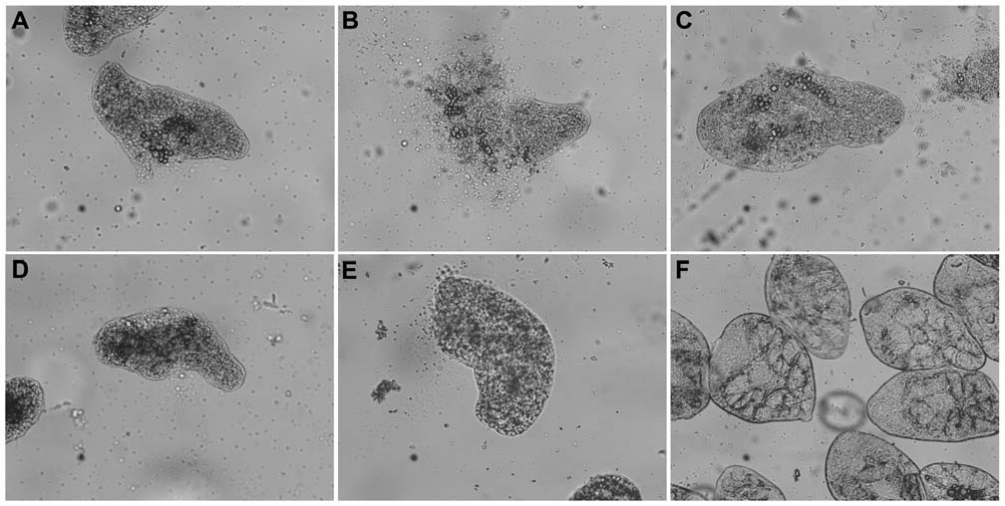
Effect of TGR inhibitors on *F. hepatica* newly excysted juvenile, tested at 20 µM during 48 h. a. **2** (oxadiazole *N*-oxide), b. **1** (oxadiazole *N*-oxide), c. **4** (oxadiazole *N*-oxide), d. **61** (quinoxaline), e. **50** (thiadiazole), f. control.

We tested the TGR inhibitors that killed the *E. granulosus* protoscoleces *in vitro* on the trematode parasite *F. hepatica*. The 12 active compounds (and the two controls, **19**, and **42**) were tested *in vitro* on newly excysted juveniles (NEJ) at 20 µM; the results are shown in **Table 4**. Similarly to the results obtained with *E. granulosus* protoscoleces, the control compounds had no effect, and most of the compounds tested (except a furoxan and a couple of benzofuroxans, **5**, **22**, and **43**) killed more than 80% NEJ. Overall, *F. hepatica* NEJ were more sensitive than *E. granulosus* protoscoleces to TGR inhibitors when examined at 20 µM. Similar to what was observed with *E. granulosus* protoscoleces, compound **4** killed NEJ with similar efficacy to most active TGR inhibitors. Remarkably, the thiadiazole identified as TGR inhibitor, **50**, was also effective *in vitro* against a trematode parasite. Of the five benzofuroxans assayed, three (**23**, **24** and **25**) were very effective in killing NEJ; two of the active ones contained the phenylsulfonyl (phenylsulfinyl) moiety, also present in some active furoxans and the identified thiadiazole. **Figure 7** shows NEJ treated with inhibitors and control compounds. Lack of parasite material precluded testing the most active compounds at lower concentrations during longer periods of exposure to the compounds.

**Table 4:** *F. hepatica* newly excysted juveniles viability *in vitro* after 48 h incubation with TGR inhibitors at 20 µM.

Compound	NEJ viability (%) at 20 µM
**2**	0
**3**	0
**4**	0
**1**	14
**5**	80
**25**	10
**23**	16
**24**	19
**22**	66
**43**	85
**50**	18
**61**	12
**19**	100 (control)
**42**	100 (control)

A control with vehicle alone (DMSO) was also included and did not affect NEJ viability.

### Identified inhibitors are not toxic to human lymphocytes at the larvicidal concentrations

To examine if most active compounds are toxic to human primary cells we performed *in vitro* culture of PBMC with these compounds at concentrations ranging from 10 to 80 µM and tested cell viability after 96 hours of culture. Only marginal toxicity was observed at 20 µM (above 92% viability). Compounds were still tolerated at 80 µM concentrations and viability ranged from 49 to 80% at this concentration after 96 hours of incubation. Additional citotoxicity data using mammalian cell lines are provided for all active compounds in **Text S1**.

## Discussion

Flatworm infections are chronic and debilitating diseases and there is a very restricted set of drugs and a few rational drug targets. The identification of novel drugs, in particular if they target the parasite by a different mechanism, remains as an important challenge in medicinal chemistry. TGR is a promising target for drug screening for flatworm infections. Two features make this enzyme particularly attractive: it is a drugabble enzyme and its inhibition leads to disruption in redox homeostasis of flatworm parasites. Based on these premises we selected, from previously synthesized products in our chemical library, a range of candidate TGR inhibitors, based on literature mining and the reactive nature (nucleophilicity) of the selenocysteine residue of TGR. The selection included compounds belonging to different structural families: oxadiazole *N*-oxide or furoxan (21), benzofuroxan (28), thiadiazole (11), quinoxaline (3), nitrooxy-derivative (1) and oxathiazole (1) (see **Table S1** and references therein).

**Figure 8:**
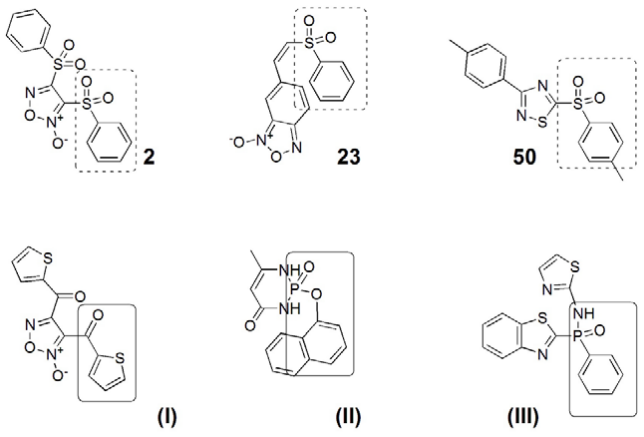
Structures of the most potent compounds described herein (2, 23, and 50) and several previously described TGR inhibitors ((I), (II), and (III)) ([13], [14]). Structural motives are marked.

A first interesting observation was that a good selection of compounds was accomplished: 14 out of 65 selected compounds inhibited both TR and GR activities of TGR. We identified several oxadiazole *N*-oxides and one quinoxaline as TGR inhibitors. These families of compounds have previously been identified as inhibitors of *S. mansoni* TGR and as drug hits for schistosomiasis. Therefore our results validate and expand previous results [12], [13], [14]: we showed that these compounds inhibited another fluke TGR (*F. hepatica*), and, more importantly, they also inhibited a tapeworm TGR (*E. granulosus*). Thus, these two families of compounds can be considered as drug hits for flatworm parasites, and not only for flukes. Another important result of our study is the identification of a thiadiazole (**50**) as a potent inhibitor of both fluke and tapeworm TGRs, indicating that this family of compounds deserves further studies. It is noteworthy that only 1 out of 11 thiadiazoles inhibited TGR, and therefore the possibility of thiadiazole as a true drug hit remains to be further explored. A striking finding of our studies is that the phenylsulfonyl/phenylsulfinyl) moiety correlated very well with TGR inhibition. Furthermore, active compounds belonging to different families -oxadiazole *N*-oxides **2** and **5**, benzofuroxans **23** and **24** (the related sulfinyl-moiety in the latter case) and thiadiazole **50**- possessed this group, suggesting that it may be a new pharmacophore. Remarkably, two of them were among the best three TGR inhibitors and parasite killers found in our study. In this sense, and taking into account the chemical structures of previously described TGR-inhibitors (i.e. compounds **(I)–(III)**, **Figure 8** ([13], [14]) one could speculate that the scaffold aryl-X = O, where X is P, S, or C, plays a relevant role in the enzymatic inhibition.

Since a relatively large number of oxadiazoles were examined in this screening, our results also shed light on the substituents on the heterocycle ring that may enhance or disfavor inhibition: C3 and C4 positions of the heterocycle are key to optimize inhibitor activity. Indeed, we found that a methyl substituent in C3 of the ozadiazole ring and very large substituents as well as aliphatic/hydrophilic substituent in C4 appeared to be detrimental to inhibition. Similar observations were made by Rai *et al.* [12]. In contrast, the presence of an electrophilic/electron-withdrawing group as a substituent in C3 favored inhibition. Similarly, a phenyl substituent in C4 favors inhibition, probably as an additional electron-withdrawing group that increases the reactivity of the furoxan system. The benzofuroxans represented the other large family examined (28 compounds). Although none of these compounds was as active as oxadiazoles, active benzofuroxans were, as in the case of furoxans, those with the presence of an electrophilic/electron-withdrawing group as benzo-substituent. The existence of a SAR pattern supported the idea that the hits were not random, and that they represent promising hit/lead structures for the development of anti-parasitic drugs. The high attrition rates observed in HTS of antiparasitic compounds is sometimes related to the lack of correlation between enzyme inhibition and cell activity. One main reason for this is dubious validation status of the target enzyme. Herein, we showed that hit compounds found in an *in vitro* TGR assay displayed a good correlation with antiparasitic activity, supporting TGR as a valid target in the development of drugs against tapeworm and fluke parasites.

For all inhibitors the percentage of inhibition found for *F. hepatica* and *E. granulosus* TGRs correlated well between both, fluke and tapeworm, enzymes. More importantly, in both cases TGR inhibition correlated very well with the *in vitro* assays using *E. granulosus* protoscoleces and *F. hepatica* NEJ: 10 of the identified inhibitors effectively killed parasites *in vitro*. Noteworthy is the fact that the most effective TGR inhibitors were those that killed parasites at lower doses. The consistency of the results strongly indicates that, in all likelihood, the antiparasitic effect observed for the compounds is due to inhibition of this essential enzyme. An exception to this trend is compound 4, which is not within the most potent inhibitors of *E. granulosus* TGR, but very effective in killing larval worms. Indeed, this compound has been found to be a more potent oxadiazole N-oxide, due to increased nitric oxide release [12], suggesting that this mechanism contributes to its toxicity. It is interesting to highlight that compounds 1, 2 and 3 showed an excellent correlation between enzyme inhibition and parasite killing. In this context, it is relevant to emphasize that these three compounds were found to slowly and irreversibly bind TGR. Thus, our results suggest that nitric oxide release and nitrosylation may play a role in their efficacy as TGR inhibitors and parasite killers. Finally, it should be mentioned that other mechanism(s) different form NO release could lead to slow and nearly irreversible inhibition of TGR as illustrated by the strong inhibition displayed by the identified thiadiazole substituted with the phenylsulfonyl moeity.

Our results reinforce the concept that the redox metabolism of flatworm parasites is particularly susceptible to destabilization, and that the TR module of TGR is a “druggable” target that leads to redox unbalance in flatworms. Specifically we showed that furoxans and quinoxalines are drug hits not only for flukes but also for tapeworms, and identified new drug hits for both classes of flatworm parasites. Since the biochemical scenario of flatworm parasites is very similar regarding the thiol redox-dependent pathways [9], [10], [11], [26], our results highlight that TGR inhibitors have broad applications for the control of a wide range of neglected diseases.

## Supporting Information

Text S1 **Information about toxicity of inhibitors of TGR.** Toxicity profiles have previously been analyzed *in vitro* on mammalian cells for the compounds analyzed *in vitro* against *E. granulosus* protoscolex and *F. hepatica* newly excysted juveniles. For some compounds mutagenicity studies by Ames' test and genotoxicity studies by comet assay were carried out.
(DOC)

Table S1 **Inhibition % of TR activity for all compounds used in the screening of TGR inhibitors.**
(DOC)
