# Supplementary material for: Identification of Thioredoxin Glutathione Reductase Inhibitors That Kill Cestode and Trematode Parasites
Source: PLoS One. 2012 Apr 20;7(4):e35033. doi: 10.1371/journal.pone.0035033 (PMC3335049; doi:10.1371/journal.pone.0035033)
Supplement: Text S1 — Information about toxicity of inhibitors of TGR. Toxicity profiles have previously been analyzed in vitro on mammalian cells for the compounds analyzed in vitro against E. granulosus protoscolex and F. hepatica newly excysted juveniles. For some compounds mutagenicity studies by Ames' test and genotoxicity studies by comet assay were carried out. (DOC) [file pone.0035033.s001.doc]

**Text S1: Information about toxicity of inhibitors of TGR**

Toxicity profiles have previously been analyzed *in vitro* on mammalian cells for the compounds analyzed *in vitro* against *E. granulosus* protoscolex and *F. hepatica* newly excysted juveniles. For some compounds mutagenicity studies by Ames´ test and genotoxicity studies by comet assay were carried out.

**Compound 1**

Tested against murine fibroblasts (L929 cell line), this study, see Material and Methods: 100% of survival at 80 µM.

**Compound 2**

Tested against murine fibroblasts (L929 cell line), this study, see Material and Methods: 100% of survival at 160 µM.

**Compound 3**

Tested against murine fibroblasts (L929 cell line), this study, see Material and Methods: 100% of survival at 160 µM. Additional data using V79 in *Monge A., López de Ceráin A., Ezpeleta O., Cerecetto H., Dias E., Di Maio R., González M., Onetto S., Seoane G., Suescun L., Mariezcurrena R.* *Pharmazie. 53 (1998) 758-764.*

**Compound 4**

**Data in** [*Boiani M*](http://www.ncbi.nlm.nih.gov/pubmed?term="Boiani M"%5BAuthor%5D)*,* [*Cerecetto H*](http://www.ncbi.nlm.nih.gov/pubmed?term="Cerecetto H"%5BAuthor%5D)*,* [*González M*](http://www.ncbi.nlm.nih.gov/pubmed?term="González M"%5BAuthor%5D)*,* [*Risso M*](http://www.ncbi.nlm.nih.gov/pubmed?term="Risso M"%5BAuthor%5D)*,* [*Olea-Azar C*](http://www.ncbi.nlm.nih.gov/pubmed?term="Olea-Azar C"%5BAuthor%5D)*,* [*Piro OE*](http://www.ncbi.nlm.nih.gov/pubmed?term="Piro OE"%5BAuthor%5D)*,* [*Castellano EE*](http://www.ncbi.nlm.nih.gov/pubmed?term="Castellano EE"%5BAuthor%5D)*,* [*López de Ceráin A*](http://www.ncbi.nlm.nih.gov/pubmed?term="López de Ceráin A"%5BAuthor%5D)*,* [*Ezpeleta O*](http://www.ncbi.nlm.nih.gov/pubmed?term="Ezpeleta O"%5BAuthor%5D)*,* [*Monge-Vega A*](http://www.ncbi.nlm.nih.gov/pubmed?term="Monge-Vega A"%5BAuthor%5D)*.* [*Eur J Med Chem.*](http://www.ncbi.nlm.nih.gov/pubmed?term=castellano boiani monge) *36 (2001) 771-782.*

**Using V79 cells at 20 μM.** Additional data on: Identification of oxadiazoles as new drug leads for the control of schistosomiasis. *Sayed AA, Simeonov A, Thomas CJ, Inglese J, Austin CP, Williams DL. Nat Med. 2008 14(4):407-12.*

**Compound 5**

Tested against murine fibroblasts (L929 cell line), this study, see Material and Methods: 100% of survival at 100 µM. Additional data in *Cabrera M, López GV, Gómez LE, Breijo M, Pintos C, Botti H, Raymondo S, Vettorazzi A, López de Ceráin A, Monge A, Rubbo H, González M, Cerecetto H. Drug Chem. Toxicol. 34 (2011), 285–293.*

**Mutagenicity studies by Ames test and genotoxicity studies by comet assay**

**Compound 6**

This compound is currently studied, in our lab^1^, against **J774.1 cells** finding a IC_50_= 246.0 µM.

**Compound 22**

Tested against murine fibroblasts (L929 cell line), this study, see Material and Methods: 100% of survival at 100 µM.

**Compounds 23 and 24**

**Data in** *Porcal W, Hernández P, Boiani M, Aguirre G, Boiani L, Chidichimo A, Cazzulo JJ, Campillo NE, Paez JA, Castro A, Krauth-Siegel RL, Davies C, Basombrío MA, González M, Cerecetto H. J Med Chem. 50 (2007) 6004-6015.*

**Data in vitro (J774.1 cells) and in vivo (murine model)**

**Compound 43**

**Data in** *Cabrera M, Lavaggi ML, Hernández P, Merlino A, Gerpe A, Porcal W, Boiani M, Ferreira A, Monge A, de Cerain AL, González M, Cerecetto H. Toxicol Lett. 190 (2009) 190, 140-149.*

**Mutagenicity studies by Ames test and genotoxicity studies by comet assay**

**Compound 44**

**Data in** *Castro D, Boiani L, Benitez D, Hernández P, Merlino A, Gil C, Olea-Azar C, González M, Cerecetto H, Porcal W. Eur. J. Med. Chem. 44 (2009) 5055–5065.*

**Data in vitro (J774.1 cells)**

**Compound 25**

**Data in** *Carlsson L, Helgee EA, Boyer S. J. Chem. Inf. Model. 49 (2009) 2551–2558.*

**Mutagenicity studies by Ames test**

**Compound 50**

**Data of this chemotype toxicity in** *Porcal W, Hernández P, González M, Ferreira A, Olea-Azar C, Cerecetto H, Castro A. J. Med. Chem. 51 (2008) 6150-6159.*

**Data in vitro (J774.1 cells)**

**Compound 61**

Tested against murine fibroblasts (L929 cell line), this study, see Material and Methods: 100% of survival at 100 µM.
