# Supplementary material for: Identification of Thioredoxin Glutathione Reductase Inhibitors That Kill Cestode and Trematode Parasites
Source: PLoS One. 2012 Apr 20;7(4):e35033. doi: 10.1371/journal.pone.0035033 (PMC3335049; doi:10.1371/journal.pone.0035033)
Supplement: Table S1 — Inhibition % of TR activity for all compounds used in the screening of TGR inhibitors. (DOC) [file pone.0035033.s002.doc]

**Table S1**

**% Inhibition of TR activity for all compounds used in the screening of TGR inhibitors**. **a.** Oxadiazole N-oxides assayed, **b.** Benzofuroxans assayed, **c.** Thiadiazoles assayed, **d.** Quinoxalines, nitrooxy and oxathiazole assayed.

1. **Oxadiazole *N-*oxides assayed [1,2]**.

| **Compound** | **Concentration at which each compound was tested (µM)** | **Thioredoxin reductase inhibition (%)** |
| --- | --- | --- |
| **1** | 10 | 90 |
| **2** | 10 | 82 |
| **3** | 10 | 70 |
| **4** | 10 | 39 |
| **5** | 10 | 37 |
| **6** | 10 | 33 |
| **7** | 10 | 26 |
| **8** | 10 | 21 |
| **9** | 10 | 18 |
| **10** | 10 | 17 |
| **11** | 10 | 16 |
| **12** | 10 | 12 |
| **13** | 10 | 11 |
| **14** | 10 | 9 |
| **15** | 10 | 8 |
| **16** | 10 | 8 |
| **17** | 10 | 7 |
| **18** | 10 | 3 |
| **19** | 10 | 2 |
| **20** | 1 | 28 |
| **21** | 1 | 14 |

**22 23 24 43^*^ 44^*^ 25 26 45^*^ 27 28 29 46^*^ 30 31 32 33 34 35 36 37 47^*^**

1. **Benzofuroxans assayed [3-7].**

| **Compound** | **Concentration at which was tested the compound (µM)** | **Thioredoxin reductase inhibition (%)** |
| --- | --- | --- |
| **22** | 10 | 38 |
| **23** | 10 | 38 |
| **24** | 10 | 36 |
| **25** | 10 | 30 |
| **26** | 10 | 27 |
| **27** | 10 | 22 |
| **28** | 10 | 22 |
| **29** | 10 | 22 |
| **30** | 10 | 18 |
| **31** | 10 | 18 |
| **32** | 10 | 16 |
| **33** | 10 | 15 |
| **34** | 10 | 15 |
| **35** | 10 | 13 |
| **36** | 10 | 13 |
| **37** | 10 | 10 |
| **38** | 10 | 6 |
| **39** | 10 | 6 |
| **40** | 10 | 3 |
| **41** | 10 | 3 |
| **42** | 10 | 0 |
| **43** | 1 | 35 |
| **44** | 1 | 35 |
| **45** | 1 | 25 |
| **46** | 1 | 21 |
| **47** | 1 | 8 |
| **48** | 1 | 0 |
| **49** | 1 | 0 |

1. **Thiadiazoles assayed [8].**

| **Compound** | **Concentration at which was tested the compound (µM)** | **Thioredoxin reductase inhibition (%)** |
| --- | --- | --- |
| **50** | 10 | 91 |
| **51** | 10 | 18 |
| **52** | 10 | 17 |
| **53** | 10 | 14 |
| **54** | 10 | 10 |
| **55** | 10 | 8 |
| **56** | 10 | 8 |
| **57** | 10 | 18 |
| **58** | 1 | 21 |
| **59** | 1 | 21 |
| **60** | 1 | 3 |

1. **Quinoxalines, nitrooxy-derivative and oxathiazole assayed [2,8,9,10].**

| **Compound** | **Concentration at which was tested the compound (µM)** | **Thioredoxin reductase inhibition (%)** |
| --- | --- | --- |
| **61** | 10 | 44 |
| **62** | 10 | 19 |
| **63** | 10 | 2 |
| **64** | 1 | 14 |
| **65** | 1 | 7 |

**References**

1. Cerecetto, H.; González, M.; Risso, M.; Seoane, G.; López De Ceraín, A.; Ezpeleta, O.; Monge, A.; Suescun, L.; Mombrú, A.; Bruno, A.M. Arch. Pharm. 2000, 333, 387.

2. López, G.V.; Batthyány, C.; Blanco, F.; Botti, H.; Trostchansky, A.; Migliaro, E.; Radi, R.; González, M.; Cerecetto, H.; Rubbo, H. Bioorg. Med. Chem. 2005, 13, 5787.

3. Porcal, W.; Hernández, P.; Boiani, L; Boiani, M.; Ferreira, A.; Chidichimo, A.; Cazzulo, J.J.; Olea-Azar, C.; González, M.; Cerecetto, H. Bioorg. Med. Chem. 2008, 16, 6995.

4. Castro, D.; Boiani, L.; Benítez, D.; Hernández, P.; Merlino, A.; Gil, C.; Olea-Azar, C.; González, M.; Cerecetto, H.; Porcal, W. Eur. J. Med. Chem. 2009, 44, 5055.

5. Porcal, W.; Hernández, P.; Aguirre, G; Boiani, L; Boiani, M.; Merlino, A.; Ferreira, A.; Di Maio, R; Castro, A.; González, M.; Cerecetto, H. Bioorg. Med. Chem. 2007, 15, 2768.

6. Aguirre, G; Boiani, L; Cerecetto, H.; Di Maio, R; González, M.; Porcal, W.; Thomson, L.; Tórtora, V.; Denicola, A; Möller, M. Bioorg. Med. Chem. 2005, 13, 6324.

7. Porcal, W.; Hernández, P.; Boiani, M.; Aguirre, G; Boiani, L; Chidichimo, A.; Cazzulo, J.J.; Campillo, N.E.; Páez, J.A.; Castro, A.; Krauth-Siegel, R.L.; Davies, C.; Basombrío, Ma; González, M.; Cerecetto, H. J. Med. Chem. 2007, 50, 6004.

8. Porcal, W.; Hernández, P.; González, M.; Ferreira, A.; Olea-Azar, C.; Cerecetto, H.; Castro, A. J. Med. Chem. 2008, 51, 6150.

9. Aguirre, G.; Cerecetto, H.; Di Maio, R.; González, M.; Alfaro, M.E.; Jaso, A.; Zarranz, B.; Ortega, M.A.; Aldana, I.; Monge-Vega, A. Bioorg. Med. Chem. Lett. 2004, 14, 3835.

10. Benitez, D.; Cabrera, M.; Hernández, P.; Boiani, L.; Lavaggi, M.L.; Di Maio, R.; Yaluff, G.; Serna, E.; Torres, S.; Ferreira, M.E.; Vera de Bilbao, N.; Torres, E.; Pérez-Silanes, S.; Solano, B.; Moreno, E.; Aldana, I.; López de Ceráin, A.; Cerecetto, H.; González, M.; Monge, A. J. Med. Chem. 2011, 54, 3624.
